# Negative affect impairs associative memory but not item memory

**DOI:** 10.1101/lm.032409.113

**Published:** 2014-01

**Authors:** James A. Bisby, Neil Burgess

**Affiliations:** 1 Institute of Cognitive Neuroscience, University College, London WC1N 3AR, United Kingdom; 2 Institute of Neurology, University College, London WC1N 3AR, United Kingdom

## Abstract

The formation of associations between items and their context has been proposed to rely on mechanisms distinct from those supporting memory for a single item. Although emotional experiences can profoundly affect memory, our understanding of how it interacts with different aspects of memory remains unclear. We performed three experiments to examine the effects of emotion on memory for items and their associations. By presenting neutral and negative items with background contexts, Experiment 1 demonstrated that item memory was facilitated by emotional affect, whereas memory for an associated context was reduced. In Experiment 2, arousal was manipulated independently of the memoranda, by a threat of shock, whereby encoding trials occurred under conditions of threat or safety. Memory for context was equally impaired by the presence of negative affect, whether induced by threat of shock or a negative item, relative to retrieval of the context of a neutral item in safety. In Experiment 3, participants were presented with neutral and negative items as paired associates, including all combinations of neutral and negative items. The results showed both above effects: compared to a neutral item, memory for the associate of a negative item (a second item here, context in Experiments 1 and 2) is impaired, whereas retrieval of the item itself is enhanced. Our findings suggest that negative affect impairs associative memory while recognition of a negative item is enhanced. They support dual-processing models in which negative affect or stress impairs hippocampal-dependent associative memory while the storage of negative sensory/perceptual representations is spared or even strengthened.

Emotional experiences can have long-lasting effects on memory. Real-life emotional events are generally thought to be remembered better than neutral ones (Brown and Kulik 1977; Neisser and Harsch 1992; Neisser et al. 1996). This view has been confirmed in numerous experiments assessing memory for visually presented information, such as words, pictures, or film clips. When the encoded information is emotionally arousing, memory retrieval is often facilitated (Bradley et al. 1992; Christianson 1992; Cahill and McGaugh 1995; Cahill et al. 1996; Canli et al. 2000). Despite the emotional enhancement of memory being a robust finding, many studies demonstrating this effect assess memory for individual items. Importantly, accurate memory for an event not only involves retrieving details of the people or objects involved but also the associations between those items and the context in which the event took place. The effects of emotion on associative memory have yet to be fully elucidated (for reviews, see Mather 2007; Chiu et al. 2013).

The formation of associations between individual items and their context is proposed to rely on mechanisms distinct from those involved in memory for single items (Gardiner 1988; Jacoby 1991; Yonelinas 2002). The hippocampus, a structure critical for episodic memory, is thought to play an essential role in associative memory processes, binding together items and the context in which they were encountered within a spatially coherent representation (O'Keefe and Nadel 1978; Cohen and Eichenbaum 1993; Burgess et al. 2002; Davachi 2006; Eichenbaum et al. 2007). Retrieval of a single item from memory does not necessarily require the retrieval of contextual details or item associations and may be supported by extrahippocampal areas such as perirhinal cortex (Aggleton and Brown 1999; Davachi 2006; Barense et al. 2007; Eichenbaum et al. 2007; Murray et al. 2007; Montaldi and Mayes 2010).

Although enhanced memory for emotional items has been shown to involve the amygdala (Hamann et al. 1999; Phelps and LeDoux 2005; Phelps 2006), the specific effects of emotion on hippocampal-dependent memory remain unclear. Amygdala recruitment during emotional arousal is proposed to modulate other medial temporal lobe (MTL) structures to facilitate encoding and consolidation (McGaugh 2000; Dolcos et al. 2004). However, when the level of stress and arousal is high, hippocampal function can be down-regulated (Kim and Diamond 2002; Henckens et al. 2009; Schwabe and Wolf 2012). Indeed, some theoretical models propose that emotional experiences might weaken context-dependent memory, resulting in impaired associations between items and their context (Jacobs and Nadel 1985, 1998; Brewin et al. 2010).

Findings from the source memory literature support the view that emotion might differentially interact with item and associative memory. Retrieval of source information associated with an item, such as an object presented in the periphery of the screen or the color of a border surrounding an item at study, is impaired when the item was emotionally arousing (Kensinger and Schacter 2006; Touryan et al. 2007; Mather and Knight 2008; Pierce and Kensinger 2011; Rimmele et al. 2011). In contrast, when associations at encoding can be incorporated within the item representation, such as the color of a presented word, memory for the associated information is enhanced by an emotional item (Doerksen and Shimamura 2001; D'Argembeau and Van der Linden 2004; MacKay and Ahmetzanov 2005; Mather and Nesmith 2008; Nashiro and Mather 2011; Schmidt et al. 2011). Taken together, these findings suggest that emotion can enhance item memory, while impairing associations between items.

A recent study by Madan et al. (2012) also suggests differential effects of emotion on associative vs. item memory. In their study, participants were presented with neutral and negative word pair associates. This design provides all combinations of pairs (pure neutral, pure negative, mixed neutral–negative). To control for findings that might be explained purely by attentional accounts (e.g., Easterbrook 1959; Christianson 1992; Reisberg and Heuer 2004), this study presented each word from a pair sequentially rather than simultaneously. At test, participants were presented with a neutral or negative word as a cue and instructed to retrieve the associated neutral or negative target. Overall, their results demonstrated that associative memory was impaired across all conditions that included a negative word. However, they also found that the retrieval of a negative target was boosted relative to retrieving a neutral target from a negative cue. This suggests that the relative increase in memory for an emotional associate might be solely driven by an enhanced representation of the negative item (also see Lewis et al. 2011 and Smith et al. 2004, 2005 for similar increases in memory when retrieving whether a neutral item was encoded with an emotional or neutral context). Surprisingly, no study to date has directly examined such associative memory relations for negative images and their specific context.

Here, we aimed to further examine the way in which negative affect can interact with item and associative or contextual memory. In Experiment 1, participants encoded neutral and negative items presented over background contexts. At test, participants made recognition judgments for items and, if recognized, were tested for the associated context by a four alternative forced choice recognition task. In Experiment 2, we used a similar design to that in Experiment 1, but incorporated a threat of shock manipulation, a method often used to induce increases in arousal and anxiety (Cornwell et al. 2007; Lissek et al. 2007; Grillon and Charney 2011). Participants encoded neutral and negative items presented visually over background scenes (four in total). However, prior to encoding participants were shown all background scenes and informed of the two that predicted threat and the two that predicted safety during each trial. During a threat condition, participants could receive a mild electric shock to their wrist. Memory was again tested for items and their associated context.

In a final experiment, we presented participants with paired associates, using a design similar to that of Madan et al. (2012). However, rather than using sequential presentation of neutral and negative words, as used by Madan et al. (2012), we simultaneously presented neutral and negative image pairs together at encoding and instructed participants to vividly imagine a link between the images. This provides a highly ecologically valid method in which participants imagine an event involving the presented images. All combinations of pairs were used to assess associations (pure neutral, pure negative, mixed neutral–negative). In this experiment, participants were presented at test with a neutral or negative cue and then required to retrieve the neutral or negative target item. During test, participants completed a four alternative forced choice for written descriptions of target associates. This allowed us to determine the way in which neutral and negative images differentially contribute to impairments in associative memory. Overall, we predicted an emotional arousal-induced decrease in memory for associations across our experiments. We also expected enhanced item memory, which might facilitate the accessibility of emotional item images at retrieval.

## Results

### Experiment 1

**Figure 1.**
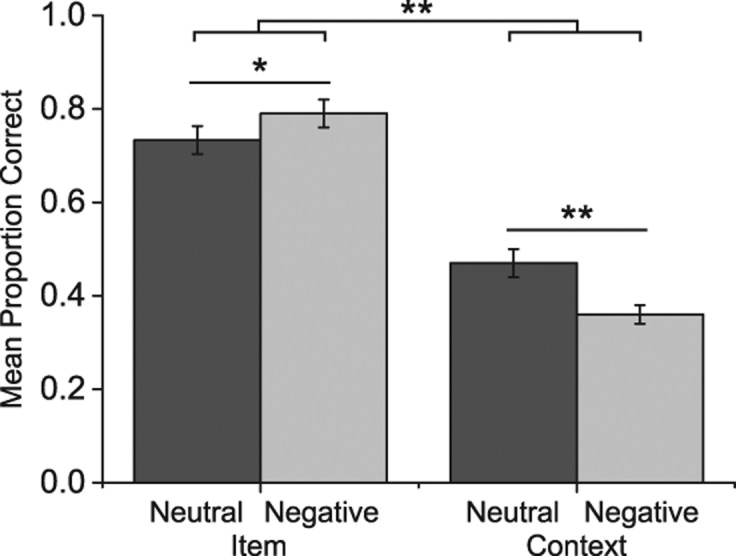
Mean proportion correct for (*left*) item recognition scores (hits minus false alarms) and (*right*) memory for contexts associated with neutral and negative items for Experiment 1. The *top* line represents the significant interaction and bars represent standard error. (*) *P* < 0.05, (**) *P* = 0.001.

We analyzed participants’ memory performance from Experiment 1 (Fig. 1) using a 2 × 2 repeated measures ANOVA with memory type (item, context) and valence (neutral, negative) as within-participant factors. Results showed a significant memory type × valence interaction (*F*_(1,17)_ = 16.36, *P* = 0.001, η_*P*_^2^ = 0.49) and a main effect of memory type (*F*_(1,18)_ = 120.35, *P* < 0.001, η_*P*_^2^ = 0.88) but not of valence (*F*_(1,18)_ = 2.00, *P* = 0.16, η_*P*_^2^ = 0.11). Further examination of the interaction showed that recognition memory performance was greater for negative items compared to neutral items (*t*_(17)_ = 2.15, *P* = 0.046, *d* = 0.52). However, the opposite pattern was observed for context memory with greater performance for retrieving contexts associated with neutral items compared to those associated with negative items (*t*_(17)_ = 4.00, *P* = 0.001, *d* = 0.95). False alarm rates for recognition at test were low for both neutral (0.03 ± 0.04) and negative items (0.04 ± 0.05) and did not differ (*P* = 0.31).

We next checked whether the observed decrease in memory for context could be explained by the plausibility of item–context association pairings at encoding. For instance, neutral item–context associations might be more plausible than negative item–context associations and thus easier to bind together. We therefore split all item–context pairings by each participant's plausibility judgment at encoding and reanalyzed associative memory data. A 2 × 2 repeated measures ANOVA on participants’ memory scores for the associated context was performed with plausibility (yes, no) and valence as within-participants factor. Analysis showed no plausibility × valence interaction (*F*_(1,17)_ = 0.65, *P* = 0.43, η_*P*_^2^ = 0.04) suggesting that the plausibility of item–context pairings did not mediate the observed pattern of results. Irrespective of valence, a significant main effect of plausibility was found (*F*_(1,17)_ = 140.38, *P* < 0.001, η_*P*_^2^ = 0.89) with better overall memory performance for item–context pairs judged as plausible compared to pairs judged as implausible (for analysis of item recognition performance split by plausibility, see Supplemental Material). Confidence ratings for item recognition and context showed a similar pattern of results to memory performance (see Supplemental Material for detailed analysis).

### Experiment 2

**Figure 2.**
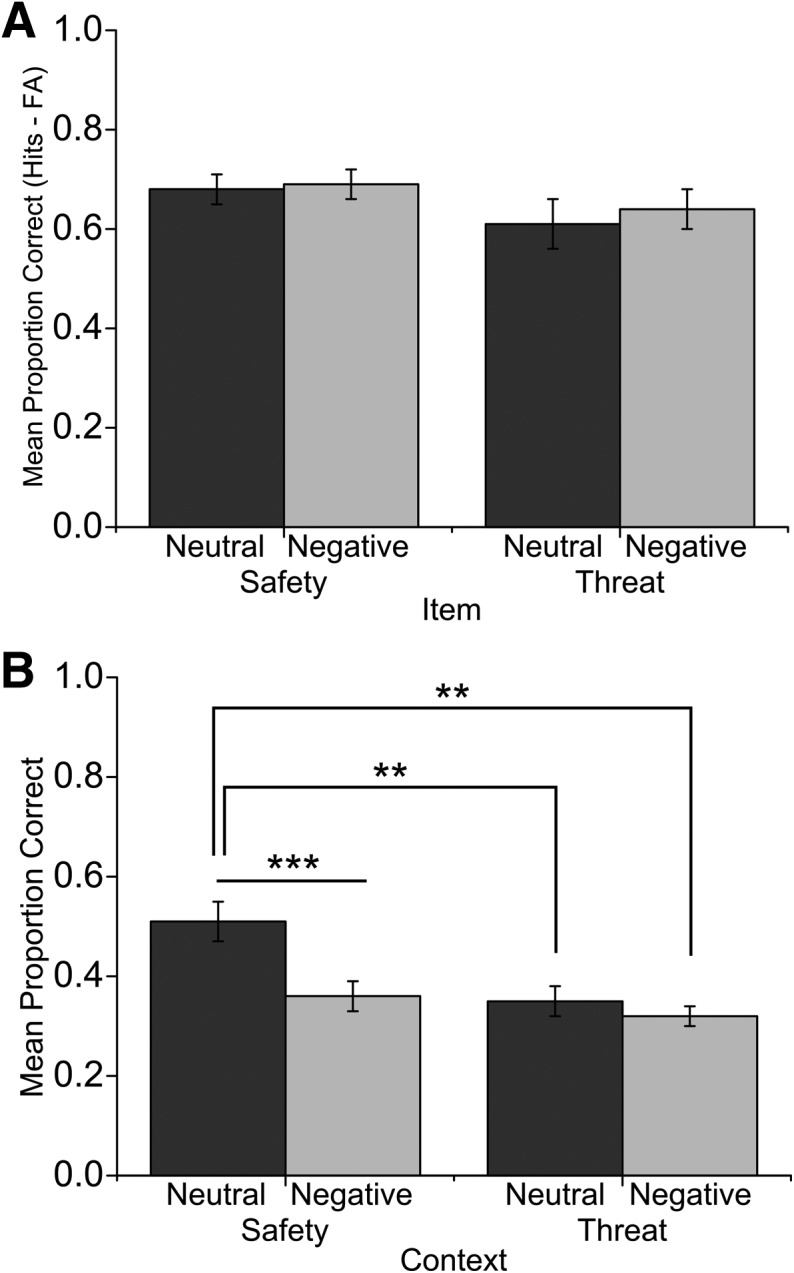
Mean proportion correct from Experiment 2 for (*A*) neutral and negative item recognition and (*B*) memory for contexts associated with neutral and negative items across safety and threat conditions. Bars represent standard error. (***) *P* < 0.001, (**) *P* < 0.01.

We performed a 2 × 2 × 2 repeated measures ANOVA on participants’ memory accuracy (Fig. 2) with memory type (item, context), condition (safety, threat), and item valence (neutral, negative) as within-participant factors. Analysis revealed a three-way interaction that was marginally significant (*F*_(1,17)_ = 4.31, *P* = 0.05, η_*P*_^2^ = 0.20). To further examine this interaction, we performed separate analyses for item and context memory performance. Analysis of recognition of neutral and negative items showed no significant interaction (*F*_(1,17)_ = 0.13, *P* = 0.72, η_*P*_^2^ = 0.01), no main effects of valence (*F*_(1,17)_ = 0.23, *P* = 0.64, η_*P*_^2^ = 0.01), and a trend of an effect for condition (*F*_(1,17)_ = 3.47, *P* = 0.08, η_*P*_^2^ = 0.17).

Examination of memory performance for contexts associated with neutral and negative items showed a significant condition × valence interaction (*F*_(1,17)_ = 8.39, *P* = 0.01, η_*P*_^2^ = 0.33) and main effects of condition (*F*_(1,17)_ = 11.68, *P* = 0.03, η_*P*_^2^ = 0.41) and valence (*F*_(1,17)_ = 10.69, *P* = 0.005, η_*P*_^2^ = 0.39). Compared to memory performance for contexts associated with neutral items in the safety condition, retrieval of context was reduced by the presence of a negative item in both safety (*t*_(17)_ = 4.77, *P* < 0.001, *d* = 1.11) and threat conditions (*t*_(17)_ = 3.76, *P* = 0.002, *d* = 1.01). In addition, the presence of threat also impaired retrieval of contexts associated with neutral items compared to safety (*t*_(17)_ = 3.76, *P* = 0.002, *d* = 0.91). All other comparisons were not significant (*P* ’s > 0.12). False alarm rates were low, although we found a significantly greater proportion of false alarms for negative item images (0.12 ± 0.08) compared to neutral (0.05 ± 0.06; *t*_(17)_ = 5.67, *P* < 0.001, *d* = 1.40). Consistent with Experiment 1, analysis of confidence ratings showed greater confidence ratings for negative compared to neutral images and the opposite pattern for context memory (see Supplemental Material).

### Experiment 3

**Figure 3.**
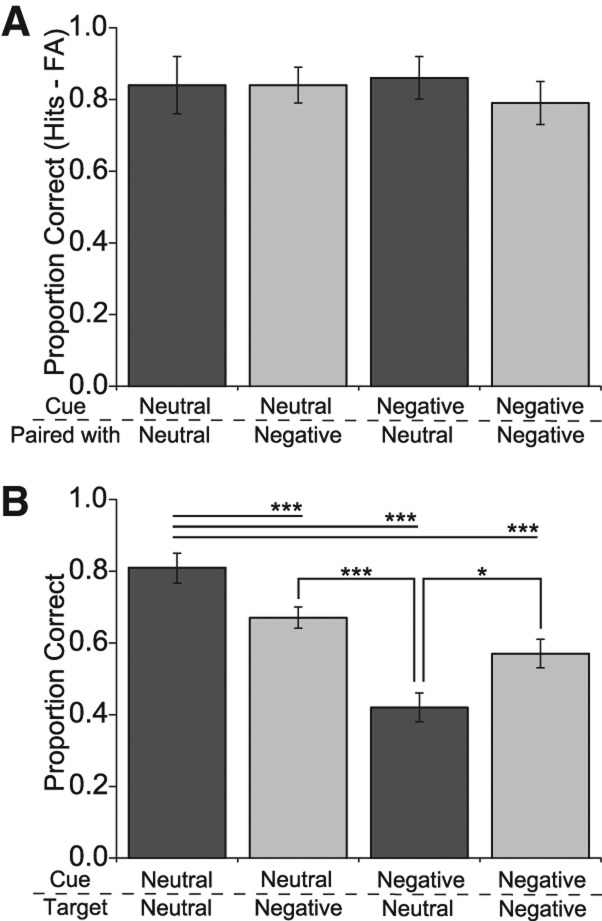
Mean proportion correct from Experiment 3 for (*A*) recognition of cue item and (*B*) retrieval of the associated target item. Bars represent standard error. (*) *P* < 0.05, (***) *P* < 0.001.

We performed an analysis of participants’ memory performance (Fig. 3) using a 2 × 2 × 2 repeated measures ANOVA with memory type (recognition, associative), cue valence (neutral, negative), and target valence (neutral, negative) as within-participant factors. This analysis showed a significant memory type × cue valence × target valence interaction (*F*_(1,14)_ = 42.91, *P* < 001, η_*P*_^2^ = 0.75). To further analyze the data we performed separate 2 × 2 ANOVAs on recognition of the item cues and on retrieval of associated targets. Analysis of item recognition performance for the cue was performed with cue valence (neutral, negative) and associate valence (neutral, negative) entered as within-participants factors. There was a trend of a main effect of target valence (*F*_(1,14)_ = 3.68, *P* = 0.08, η_*P*_^2^ = 0.21) but no significant main effect of cue valence (*F*_(1,14)_ = 2.06, *P* = 0.17, η_*P*_^2^ = 0.13) or cue × associate valence interaction (*F*_(1,14)_ = 3.08, *P* = 0.10, η_*P*_^2^ = 0.18).

Results from participants’ associative memory performance using a similar 2 × 2 ANOVA of cue and target valence (neutral, negative) showed a significant interaction (*F*_(1,15)_ = 23.72, *P* < 0.001, η_*P*_^2^ = 0.63). A main effect of cue valence was also observed (*F*_(1,15)_ = 96.44, *P* < 0.001, η_*P*_^2^ = 0.87) but not of target valence (*F*_(1,15)_ = 0.09, *P* = 0.77, η_*P*_^2^ = 0.01). Further analysis of the interaction showed that, when cued with a negative item, memory for a neutral associate target was impaired compared to neutral–neutral (*t*_(15)_ = 11.74, *P* < 0.001, *d* = 3.49), neutral–negative (*t*_(15)_ = 6.26, *P* < 0.001, *d* = 1.73), and negative–negative item pairs (*t*_(15)_ = 2.27, *P* = 0.04, *d* = 0.64). In addition, neutral–negative (*t*_(15)_ = 4.09, *P* = 0.001, *d* = 1.14) and negative–negative (*t*_(15)_ = 5.65, *P* < 0.001, *d* = 1.48) item pairs showed lower memory performance for the target associate compared to neutral–neutral pairs and also differed significantly from each other (*t*_(15)_ = 3.18, *P* = 0.007, *d* = 0.79). To summarize, reduced associative memory was observed in all conditions that included a negative item compared to performance on pure neutral item pairs. Further, for pairs that included a negative item, retrieval of the target associate was enhanced when negative relative to retrieval of a neutral target associate when cued by the negative item.

False alarm rates were low for both neutral (0.07 ± 0.08) and negative (0.05 ± 0.05) items and showed no significant differences (*t*_(14)_ = 1.05, *P* = 0.31). Analysis of confidence ratings showed lower confidence for the target associate when the cue was negative compared to neutral (see Supplemental Material for confidence rating analyses).

## Discussion

We examined the effects of emotion on item and associative memory, manipulating emotional affect through use of negative items and an anticipatory threat of shock. The main findings are: (1) negative affect reduced associative memory, as evidenced by a significant decrease in the retrieval of an associated item or context; (2) the impairing effect of negative emotion on associative memory was observed, whether induced by a negative visual stimulus or by an anticipatory threat of shock; (3) recognition memory for negative items was either enhanced or unaffected compared to recognition of neutral items; (4) in addition to a general impairment in associative memory by negative affect, retrieval of a negative target associate was improved relative to that of a neutral target (Experiment 3), perhaps reflecting enhanced item memory for negative stimuli.

Across all of our experiments, negative emotion was found to impair associative memory, a finding in accordance with previous reports (Touryan et al. 2007; Mather and Knight 2008; Rimmele et al. 2011). This robust impairment was unaffected by manipulations to the type of information to be associated (context or item), or the way in which emotional arousal was induced (negative visual images or anticipatory threat of shock). Impaired associative memory in our study contrasts with some findings reporting enhanced associative memory for negative stimuli (e.g., D'Argembeau and Van der Linden 2004; MacKay and Ahmetzanov 2005; Kensinger and Schacter 2006; Mather and Nesmith 2008). However, these studies utilize tasks in which the association directly concerns the “content” of the stimulus and so could be incorporated within the item representation. For example, remembering the color of a word (Doerksen and Shimamura 2001) or the location of the word on a screen (D'Argembeau and Van der Linden 2004), or else tests of recognition memory for an associated item rather than memory for the association itself (Knight and Mather 2009). Based on the pattern of results observed in these studies and our own, emotional stimuli might enhance within-item representations, while impairing between-item associations (see below for further discussion). In addition, impairments in associative memory for negative stimuli were observed across all experiments, whether four background scenes were used (Experiments 1 and 2) or when pairs were completely novel (Experiment 3). Therefore, we do not believe that interference from overlapping item and context pairs in Experiments 1 and 2 strongly influenced our results.

In contrast to impaired associative memory, item recognition was either enhanced (Experiment 1) or unaffected (Experiment 2; see below for further discussion) by emotional manipulation. A similar pattern of results was also observed in participants’ confidence ratings with reduced confidence for contexts associated with negative items compared to neutral items (Supplemental Figs. S2 and S3). Increased memory for negative items is also consistent with numerous previous studies (Cahill and McGaugh 1995; Cahill et al. 1996; Canli et al. 2000). Opposing results between item recognition and associative memory suggest that negative emotion might interact with distinct memory mechanisms in different ways. For instance, the formation of associations between items and context is thought to be supported by the hippocampus (O'Keefe and Nadel 1978; Cohen and Eichenbaum 1993; Aggleton and Brown 1999; Burgess et al. 2002; Davachi 2006; Eichenbaum et al. 2007). Decreased associative memory in our study highlights a particular sensitivity in hippocampal-dependent memory relating to increases in negative emotion.

The results from Experiment 3 are informative in further dissociating the way in which memory for individual items and their associations are affected by negative emotion. By including all possible combinations of neutral and negative item pairs at encoding we could examine associative memory and the way in which cue and target valence might independently contribute to memory performance. As observed throughout our experiments, the inclusion of a negative item at encoding resulted in a consistent reduction in associative memory at test (when compared to performance on pure neutral pairs). This significant decrease was observed irrespective of whether the cue or target associate was negative in valence. In addition to this impairment, performance on trials involving a negative item was affected by the valence of the target associate. When the target associate was negative, retrieval of that item was facilitated compared to trials when a negative cue was presented and participants were required to retrieve a neutral target associate. The relative increase in item retrieval of a negative target suggests an increase in an item's accessibility from memory relating to its emotional status. An increase in negative target retrieval, in combination with reduced hippocampal-dependent associative memory, might reflect recruitment of memory structures outside of the hippocampus and their modulation through emotion (see below for further discussion). Results from Experiment 3 also demonstrated discrete differences in participants’ confidence ratings dependent on the valence of the presented cue (Supplemental Fig. S5). When the cue was negative, confidence for the target associate was lower than when the cue was neutral. Presentation of a negative cue might further impinge on participants’ certainty of memory strength during associative memory retrieval.

In addition to demonstrating a general reduction in associative memory relating to negative items, Experiment 2 also provided evidence that negative affect generated by anticipatory threat can disrupt memory in a similar way. In Experiment 2, retrieval of contextual information associated with neutral items was reduced when the context predicted an aversive shock. Previous studies have used a threat of shock manipulation to assess increases in arousal and anxiety on cognitive performance (Cornwell et al. 2007; Lissek et al. 2007; Grillon and Charney 2011) although, to our knowledge, none to date have examined its differential effects on item and associative memory. Anticipatory shock therefore impaired the encoding of contextual information associated with neutral items in a similar way to memory impairments observed for negative items. Further, anticipatory shock did not affect item memory, with recognition performance showing a consistent pattern across threat and safety conditions.

How might negative affect interact with memory processes to specifically enhance item memory, while impairing memory for associations? Dual processing accounts of memory propose functionally dissociable mechanisms that support item recognition and memory for associations (Rugg and Yonelinas 2003; Diana et al. 2007; Eichenbaum et al. 2007). That is, increased familiarity of items to support recognition relies on perirhinal cortex (Aggleton and Brown 1999; Davachi 2006; Barense et al. 2007; Eichenbaum et al. 2007; Murray et al. 2007; Montaldi and Mayes 2010), whereas associative memory is supported by the hippocampus (O'Keefe and Nadel 1978; Cohen and Eichenbaum 1993; Burgess et al. 2002; Davachi 2006; Eichenbaum et al. 2007). Our results are compatible with this view in that item and associative memory were affected in opposite directions. Speculatively, negative emotion might enhance item memory through amygdala-dependent processes and modulation of other MTL structures, such as perirhinal cortex (for similar views, see Mather 2007; Murray and Kensinger 2013). In further support of this link, a recent study in rodents demonstrates a potential role for the amygdala in recognition memory, with damage to this structure found to reduce familiarity-type recognition of nonemotional items, while recollection-type processes were spared (Farovik et al. 2011). In contrast to enhanced recognition of emotional stimuli, reduced associative memory in our study suggests a potential disruption to hippocampal processing.

Our findings are also in line with a “dual-representation” model of emotional memory (Jacobs and Nadel 1985, 1998; Brewin et al. 2010). This account proposes that increases in stress and arousal can differentially affect memory representations. Lower level sensory/perceptual representations can be strengthened at encoding through recruitment of structures involved in emotional responses. In contrast, contextual representations are weakened or impoverished due to the emotional response and down-regulation of the hippocampus. Enhanced memory for negative items in combination with reduced associative memory in our study is consistent with such a model.

In Experiment 2, anticipatory threat did not enhance item memory, suggesting that the additional increase in arousal from threat did not interact with item memory processing. Sensory details of the negative item itself therefore seem to play an important role in strengthening sensory/perceptual representations to facilitate item memory. We also note that an increase in recognition of negative items was not observed in Experiments 2 and 3. A lack of enhancement in Experiment 3 can be explained by the high recognition performance and possible ceiling effects across conditions. In Experiment 2, the use of a threat of shock procedure might have contributed to the absence of increased recognition memory for negative items. Threat of shock during encoding, as predicted by the background scene, could potentially override the arousal effects that would be normally triggered by the negative stimuli presented as an item. Results from our skin conductance data partially support this view. Greater skin conductance responses were observed during trials that predicted a threat of shock compared to safety, although no differences were observed between neutral and negative items under threat. In contrast, examination of skin conductance during safety trials revealed significantly greater increases in skin conductance responses for negative items compared to neutral items. However, we also report a significant increase in false alarm rates for negative items. When item recognition data were reanalyzed without correcting for false alarm rates, a significant increase in recognition memory for negative items was observed (see Supplemental Material). Although previous research has shown increases in false alarms rates for negative images compared to neutral (Maratos et al. 2000; Dougal and Rotello 2007), it is interesting to note that many investigations of emotional memory do not often correct for accuracy (hits minus false alarms; see Murty et al. 2011 for further discussion). The combination of increased false alarm rates and recognition performance for negative items in our study suggests a potential response bias for negative information.

Overall, our results complement a recent study showing associative memory reductions to negative words. Madan et al. (2012) sequentially presented participants with all combinations of neutral and negative word pairs. As in our study, they showed impaired associative memory when a pair included a negative word, and a relative increase in retrieval of a negative target word. We extend these results by showing a similar pattern of results, with larger effect sizes, when using simultaneous presentation of neutral and negative pictures to produce an ecologically valid experience in which participants imagine an event (i.e., more comparable to experiencing a negative event). In addition, we demonstrate that reduced associative memory is also observed when associating negative items with their context (Experiment 1) and when negative affect was induced by an anticipatory threat—i.e., independently of the items themselves (Experiment 2).

General explanations for observed impairments in associative memory have focused on the effects of emotional salience on directed attention. For example, attentional accounts propose that emotional aspects of a scene capture attentional resources to enhance memory for the emotional item at the expense of nonemotional peripheral information (e.g., Easterbrook 1959; Christianson 1992; Reisberg and Heuer 2004). Although increased attention toward an emotional stimulus might play some role in reduced memory for associations (e.g., Maddox et al. 2012), we highlight that such accounts are insufficient. By using a threat of shock, we were able to generate an increase in arousal that was predicted by contextual information and not specific to the valence of item images. This manipulation resulted in reduced associative memory despite the predictive value of context that would be expected to capture participants’ attention. Results from our skin conductance data further demonstrate that the threat of shock manipulation was successful in negating some of the potential effects of emotional items on attentional resources. That is, greater increases in skin conductance responses during threat trials compared to safety suggests that participants did attend to background scenes during encoding. This increase in attention to background scenes would be expected to facilitate memory for context under divided attention accounts rather than resulting in impaired performance. We also observed impaired associative memory using paired associates (Experiment 3) when both items were negative and, thus, comparable in their attentional demands. Therefore, the observed effects of emotion on specific components of memory in our experiments cannot be explained purely by changes in attention at encoding.

In conclusion, the results from our study show a robust impairment in associative memory following emotional arousal. In contrast, memory for individual items was either enhanced or unaffected by increases in arousal. These findings demonstrate the way in which emotional arousal can have differential effects on distinct memory representations. Overall, our findings support a dual representation model whereby increases in negative affect impair memory for associations between items or between an item and its context, while the representations of negative items themselves are strengthened relative to neutral items.

## Materials and Methods

In total, 52 healthy volunteers aged 18–39 yr were recruited from the University student population. Experiment 1 included 18 participants (five males, mean age = 24.40 ± 7.10); Experiment 2, 18 participants (10 males, mean age = 26.17 ± 5.54); and Experiment 3, 15 participants (six males, mean age = 22.65 ± 4.11). The study was approved by the University College London Research Ethics Committee and participants gave signed consent before taking part in the experiment.

Neutral and negative images used in the experiments were drawn from the International Affective Pictures System (IAPS; Lang et al. 2005). Images for each experiment were taken from a pool of 180 neutral (mean valence = 5.19 ± 0.59, mean arousal = 3.47 ± 0.61) and 180 negative images (mean valence = 2.27 ± 0.60, mean arousal = 5.91 ± 0.60). Images were chosen on the basis that they depicted a central object or person with few contextual details (see Supplemental Fig. S1 for example stimuli and an illustration of the design). For Experiments 1 and 2, four background scenes were taken from the internet and included a desert scene, an arctic scene, a cityscape, and a countryside scene. Background scenes were chosen on the basis that they did not include any people or obvious objects. These scenes were used as associated contexts in the experiments.

Mild electric stimulation during Experiment 2 was delivered by a bar electrode attached to the participant's wrist of their nondominant hand using a constant voltage stimulator (Biopac Systems Inc.). Before the experiment began, a shock workup procedure was performed with shock intensity individually set by each participant to a level reported as annoying but not too painful. The shock had a duration of 200 msec and its intensity ranged from 25 V to 60 V (mean = 40.38 ± 10.25). Skin conductance responses were measured throughout the encoding phase of the task (Experiment 2) via silver/silver chloride (Ag/AgCl) electrodes attached to the participant's medial phalanges of the index and middle finger of the nondominant hand. Physiological recordings were controlled via a digital amplifier (Biopac Systems Inc.). Skin conductance responses were scored by taking the base-to-peak difference for the first waveform that occurred during the 0.5–6 sec after trial onset with a minimum response criterion of 0.02 μsec and lower responses scored as zero.

### Procedure

#### Experiment 1

Participants first performed an encoding task in which they were presented with 45 neutral and 45 negative images randomly drawn from the pool of images. For each trial, participants were presented with one of the four background scenes for a 3-sec period, after which one of the neutral or negative images appeared in the center of the screen in combination with but not completely covering the background scene and both remained on the screen for a further 3 sec. Participants were informed that they needed to pay attention to both the foreground item image and the background scene. While the image–context combination was displayed, participants were instructed to make a decision on whether they thought the event in the foreground item image could have occurred in the background scene. Participants were required to respond by button press for “yes” or “no” while the image–scene combination was on screen. Image presentation was followed by a 5-sec inter-trial interval before the next trial began.

Participants returned 24 h later for a surprise memory test of the image–context combinations they had viewed. At test, all 90 of the original foreground item images were mixed with a further 90 from the total pool of images (45 neutral and 45 negative). For each test trial, one of the foreground item images was presented on the screen and participants were instructed to make an OLD or NEW response. After responding, they gave a confidence rating on their decision from 1 (not at all confident) to 6 (very confident). If an image was recognized as old, participants were then shown the four background scenes and were required to make a decision on which scene had been originally presented with the recognized image. They were instructed to give a further confidence rating from 1 to 6 on their decision.

#### Experiment 2

The procedure for Experiment 2 was similar to that described in Experiment 1 apart from the addition of a threat of shock manipulation. Before encoding, participants were shown each of the four background scenes to be used during the experiment. For two of the scenes, participants were informed that whenever an encoding trial involved one of these scenes, there was a threat they might receive a mild electric shock. Participants were also told that the other two backgrounds scenes predicted safety and they would never receive shock during a trial which featured a safety scene. Threat and safety backgrounds were counterbalanced across participants. Shock was randomly paired with 10% of threat trials and occurred at termination of the encoding trial. At encoding, trials were presented in random order and, therefore, threat and safety could alter on a trial-by-trial basis. As in Experiment 1, participants encoded neutral and negative item images (120 neutral and 120 negative) presented with background scenes. To assess participants’ level of arousal, we recorded skin conductance responses throughout the encoding phase of the task. Participants returned after 24 h for a surprise memory test, which involved the previously encoded 240 images mixed with a further 120 new images (60 neutral and 60 negative). The structure of the memory test was the same as that described in Experiment 1.

#### Experiment 3

At encoding, participants were presented with paired associates of item images consisting of 20 pure neutral, 20 pure negative, and 40 mixed neutral–negative trials. Each pair was presented next to each other on the screen for 4 sec and participants were instructed to vividly imagine a link between the two images. Left and right item image placement for neutral–negative trials was counterbalanced. After the encoding phase and a short break (∼5–10 min), participants completed a memory test for the paired associates. During test, one item image from the pair was presented to the participants and they were instructed to make an OLD/NEW judgment on the item image. Following their response, a confidence rating was made from 1 to 6. If an item was recognized as being old, participants were required to try and remember the paired associate. For this part of the test, participants were shown four written descriptions of other item images and they were required to make a response. A confidence rating was again completed for their response to the paired associate. An additional 40 neutral and 40 negative images were used as new cues during the memory test.

### Design and statistical analysis

All data from our experiments were analyzed using repeated measures ANOVA with memory type (item, context), item valence (neutral, negative), and condition (Experiment 2; safety, threat) entered as within-participant factors. Item and associative memory performance were analyzed together to determine whether emotional manipulation would result in differential effects on memory (i.e., interaction effects between emotion and memory). Recognition memory performance throughout our experiments was corrected for false alarm rates by subtracting the number of false alarms for each valence of new items from the total number of old item correct responses. Effect sizes were calculated for ANOVA using partial η^2^ and Cohen's *d* was computed for paired samples comparisons. For analyses of confidence ratings for all experiments see Supplemental Materials.
